# Safety of a dose-escalated pre-workout supplement in recreationally active females

**DOI:** 10.1186/s12970-015-0074-y

**Published:** 2015-02-21

**Authors:** Roxanne M Vogel, Jordan M Joy, Paul H Falcone, Matt M Mosman, Michael P Kim, Jordan R Moon

**Affiliations:** MusclePharm Sports Science Institute, MusclePharm Corp., 4721 Ironton St. Building A, Denver, CO 80239 USA; Metropolitan State University, Denver, CO USA; Department of Sports Exercise Science, United States Sports Academy, Daphne, AL USA

**Keywords:** Pre-workout, Safety, Health, Female

## Abstract

**Background:**

Pre-workout supplements (PWS) have increased in popularity among athletic populations for their purported ergogenic benefits. Most PWS contain a “proprietary blend” of several ingredients, such as caffeine, beta-alanine, and nitrate in undisclosed dosages. Currently, little research exists on the safety and potential side effects of chronic consumption of PWS, and even less so involving female populations. Therefore, the purpose of the present study was to examine the safety of consuming a dose-escalated PWS over a 28-day period among active adult females.

**Methods:**

34 recreationally active, adult females (27.1 ± 5.4 years, 165.2 ± 5.7 cm, 68.2 ± 16.0 kg) participated in this study. Participants were randomly assigned to consume either 1 (G1) or 2 (G2) servings of a PWS daily or remain unsupplemented (CRL) for a period of 28 days. All were instructed to maintain their habitual dietary and exercise routines for the duration of the study. Fasting blood samples, as well as resting blood pressure and heart rate, were taken prior to and following the supplementation period. Samples were analyzed for hematological and clinical chemistry panels, including lipids.

**Results:**

Significant (p < 0.05) group by time interactions were present for absolute monocytes (CRL −0.10 ± 0.10; G1 + 0.03 ± 0.13; G2 + 0.01 ± 0.12×10E3/uL), MCH (CRL −0.13 ± 0.46; G1 + 0.36 ± 0.52; G2 -0.19 ± 0.39 pg), creatinine (CRL 0.00 ± 0.05; G1 -0.06 ± 0.13; G2 -0.14 ± 0.08 mg/dL), eGFR (CRL −0.69 ± 5.97; G1 + 6.10 ± 15.89; G2 + 14.63 ± 7.11 mL/min/1.73), and total cholesterol (CRL −2.44 ± 13.63; G1 + 14.40 ± 27.32; G2 -10.38 ± 15.39 mg/dL). Each of these variables remained within the accepted physiological range. No other variables had significant interactions.

**Conclusion:**

The present study confirms the hypothesis that a PWS containing caffeine, beta-alanine, and nitrate will not cause abnormal changes in hematological markers or resting vital signs among adult females. Although there were statistically significant (p < 0.05) group by time interactions for absolute monocytes, MCH, creatinine, eGFR, and total cholesterol, all of the results remained well within accepted physiological ranges and were not clinically significant. In sum, it appears as though daily supplementation with up to 2 servings of the PWS under investigation, over an interval of 28 days, did not adversely affect markers of clinical safety among active adult females.

**Electronic supplementary material:**

The online version of this article (doi:10.1186/s12970-015-0074-y) contains supplementary material, which is available to authorized users.

## Background

Nutrient timing refers to the methodical, timed ingestion of carbohydrate, protein, fat and other dietary supplements either before, during, or after physical activity [1]. Supplementation during the period immediately preceding physical activity has become an increasingly popular strategy among competitive and recreational athletes alike as a means of improving performance [2]. In response to this trend, manufacturers have developed pre-workout supplements (PWS), which typically combine caffeine with any number of purported ergogenic substances, such as beta-alanine, nitrate, and amino acids. As the number of PWS available on the market grows, each containing their own “proprietary blend” of active ingredients, it must be determined which, if any, are safe for chronic consumption. This becomes particularly important as concerns have arisen over the concept of proprietary blends, namely the fact that the Food and Drug Administration does not monitor the amounts of ingredients used in these blends or the accuracy of product labeling by manufacturers [2].

Caffeine is one of the most commonly found ingredients in PWS. An extensive amount of scientific literature exists on the ergogenic properties of caffeine [3-5]. According to the International Society of Sports Nutrition’s position stand on caffeine and performance, it is most effective when consumed in low to moderate doses, about 3–6 mg per kilogram bodyweight, 30–60 minutes prior to exercise [4]. Caffeine has been shown to improve performance in endurance events and time-trials, improve cognitive function and alertness, and delay the onset of fatigue during exhaustive exercise [3,5]. Moreover, caffeine anhydrous, which is frequently used in PWS, has been shown to have greater ergogenic effects than caffeine ingested in the form of coffee, tea, or cola [4].

Beta-alanine (BA), another common ingredient in PWS, is an amino acid which serves as a rate-limiting precursor to carnosine in skeletal muscle [6]. Carnosine’s suggested mechanism of action may be to buffer hydrogen ions during exercise, thereby influencing intracellular muscle pH, and ultimately increasing work capacity [7]. In a recent review of the literature by Quesnele et al. [8], the authors concluded that although there is evidence to suggest that BA supplementation enhances athletic performance, the safety of its use remains unclear, and there is a general under-reporting of its side effects in the literature.

Despite the existing literature pertaining to individual ingredients contained in PWS and the growing number of studies that address multi-ingredient PWS specifically, we are unaware of any published reports examining the safety of PWS in a solely female population. Therefore, the purpose of the present study was to examine the safety of chronic consumption of a PWS over a 28 day period among active adult females. We hypothesized that daily PWS supplementation would not produce abnormal changes in hematological or metabolic safety markers or resting vital signs.

## Methods

### Experimental design

In a dose-escalated, simple randomized design, 34 subjects were randomly assigned to control (CRL, n = 16; 27.1 ± 5.9 y, 166.2 ± 4.0 cm, 65.2 ± 12.9 kg), 1 serving (G1, n = 10; 24.9 ± 3.9 y, 164.7 ± 5.8 cm, 72.4 ± 23.3 kg), or 2 serving (G2, n = 8; 29.6 ± 5.8 y, 163.8 ± 8.8 cm, 69.0 ± 11.6 kg) groups via random number generation by the investigators and asked to remain unsupplemented, or consume either 1 or 2 servings, respectively, of a pre-workout formula (Fitmiss Ignite™, MusclePharm Corp., Denver, CO) every day for 28 days. The pre-workout formula contained 1 g of carbohydrate, 23 mg of Calcium, and 5,700 mg of a proprietary blend consisting of beta-alanine, choline bitartrate, L-tyrosine, glycine, taurine, L-carnitine, beetroot extract, hawthorn berry powder, agmatine sulfate, caffeine anhydrous, and huperzine A. The supplement was analyzed by a third party (Eurofins Supplement Analysis Center, Petaluma, CA) and verified to contain all of the ingredients on the label. Subjects were instructed to consume 1–2 level scoop(s) of the supplement with 12 oz water per scoop either 30 minutes prior to exercise or at the same time of day on rest days. Compliance was monitored using supplement consumption logs, as well as by weighing supplement containers before and after the supplementation period. A total of 38 subjects were initially recruited for this study. From G1, one subject discontinued the study due to noncompliance, and from G2, three subjects discontinued due to noncompliance. The CRL group contained more total participants, as CRL group data was added from a previously conducted study which featured a design exactly identical to the present study. Participants completed the study with an average supplementation compliance of 94.6% for G1 and 100% for G2. Blood draws were taken prior to and following the supplementation period. Approval for the human subject protocol was obtained from MusclePharm Sports Science Institute’s IRB, and subjects were provided with written informed consent documents prior to participation in the study.

### Participants

34 recreationally active female adults (27.1 ± 5.4 years, 165 ± 5.7 cm, 68.2 ± 16.0 kg) participated in the study. Recreationally active was defined as habitually participating in moderate to vigorous physical activity on three or more days a week for a duration of thirty minutes or more. Subjects were required to be non-smokers, free of any disease or disorder which may have produced confounding effects, and have abstained from taking any other pre-workout supplements for one month prior to the beginning of the study. Exclusion criteria included having a significant history or current presence of a treated condition, such as high blood pressure (≥140 mmHg systolic and/or ≥90 mmHg diastolic), tachyarrhythmia, or heart, kidney, or liver disease, or any contraindication to physical activity. Also excluded from the study were participants whose willingness or ability to comply with the study protocol was uncertain. Eligibility was determined upon evaluation of pre-participation health history, exercise, and supplementation screening questionnaires. A caffeine usage questionnaire was given as part of the pre-participating screening process, with average self-reported caffeine consumption prior to study being 131 mg/day for G1 and 269 mg/day for G2. Subjects were instructed to maintain their habitual dietary and exercise routines, and to not take any additional supplements during their participation in the study.

### Measurements

All measurements were taken prior to and following the 28-day supplementation period in a quiet, temperature controlled private office. Upon arrival at the office, subjects were instructed to remain seated quietly for 15 minutes before resting vital signs, height, and weight were taken. Subjects then submitted a blood sample in the fasted state. All blood draws were performed in the morning to prevent diurnal variations by a trained phlebotomist via venipuncture. Samples were analyzed for comprehensive metabolic panels, complete blood counts and lipid profiles by an external laboratory (Laboratory Corporation of America, Denver, CO). Variables recorded from blood analysis consisted of white blood cell count (WBC), red blood cell count (RBC), hemoglobin, hematocrit, mean corpuscular volume (MCV), mean corpuscular hemoglobin (MCH), mean corpuscular hemoglobin concentration (MCHC), red blood cell distribution width (RDW), platelets (absolute), neutrophils (percent and absolute), lymphocytes (percent and absolute), monocytes (percent and absolute), eosinophils (percent and absolute), basophils (percent and absolute), serum glucose, blood urea nitrogen (BUN), creatinine, estimated glomerular filtration rate (eGFR), BUN:creatinine, sodium, potassium, chloride, carbon dioxide, calcium, protein, albumin, globulin, albumin:globulin, bilirubin, alkaline phosphatase, aspartate aminotransferase (AST), alanine aminotransferase (ALT), total cholesterol, triglycerides, high density lipoprotein (HDL) cholesterol, and low density lipoprotein (LDL) cholesterol. Inter-test reliability results from 12 men and women measured up to one week apart at the aforementioned laboratory resulted in no significant differences for any of the variables noted above from day-to-day (p > 0.05) and an average inter-test Coefficient of Variation (CV) of 6.9%.

### Statistical analyses

Data was analyzed using a 3×2 repeated measures ANOVA model for all group, time, and group by time interactions. A Bonferroni post-hoc analysis was used to locate differences. Shapiro-Wilk tests were used to determine normality of the data. The Minimal Difference (MD) needed to be considered real was determined using the method previously described by Weir [9]. Data are presented as means ± standard deviation. All data were analyzed using Statistica software (Statsoft, Version 10).

## Results

Significant group by time interactions were present for absolute monocytes (p < 0.05), wherein CRL decreased relative to G1 and G2. Absolute monocytes had a normal distribution at baseline (p = 0.07), yet the distribution was positively skewed (p < 0.05) after the supplementation period. Significant group by time interactions were observed with MCH (p < 0.05), with G1 increasing relative to CRL and G2. Significant group by time interactions were detected for creatinine (p < 0.05), with G2 decreasing relative to CRL. Significant group by time interactions were noted for eGFR (p < 0.05), with G2 increasing relative to control. Significant group by time interactions were also present for total cholesterol (p < 0.05), G1 increasing relative to CRL and G2. Total cholesterol was positively skewed at baseline (p < 0.05), and at post-supplementation, it became normally distributed (p = 0.99). MCH and eGFR were normally distributed (p > 0.05) at both time points, and creatinine was positively skewed (p < 0.05) at both time points. All variables remained within the accepted physiological range at baseline and post supplementation. No other variables had significant group by time interactions. Data are presented in Additional file 1 as means ± standard deviation. Tolerability data collected from participants reported no serious adverse events. The most common reported side effects were a tingling sensation (n = 6), itchiness (n = 2), and nausea (n = 2). Other reported side effects included dizziness, lightheadedness, dry mouth, headache, a burning sensation, and diarrhea (all n = 1). Most of these effects occurred within the first several days of supplementation and subsided over time.

## Discussion

The results of the present study suggest that daily supplementation with the PWS under investigation does not appear to cause any abnormal changes in hematological and clinical chemistry/metabolic safety markers or resting vital signs in female subjects. While significant group by time interactions (p < 0.05) were observed for absolute monocytes, MCH, creatinine, eGFR, and total cholesterol, all group values remained well within the accepted physiological range and were not clinically significant. While remaining within range, unusual effects were observed between groups. For instance, the CRL group decreased relative to G1 and G2 for absolute monocytes, and for MCH and total cholesterol, G1 increased relative to both CRL and G2. Similar to total cholesterol, although not reaching significance (p > 0.05), both LDL and HDL increased in G1 but decreased in G2 over time. These findings are somewhat discrepant, since intuitively, one would think that if a lower dose increases a given parameter compared to control, then a higher dose should amplify this effect. This, however, was not the case. Such results suggest a natural variation in these clinical markers, and may not necessarily be related to supplementation. Additionally, the control group (n = 16) was larger than either of the experimental groups (G1, n = 10; G2, n = 8), so individual variations within the experimental groups had greater impact on the group mean values.

Variables that were significantly different at the group level were evaluated at the individual level to determine clinical significance. Analysis of clinical significance at the individual level was conducted using the MD statistic, which calculates the magnitude of the inter-test difference (between baseline and post-supplementation) needed to be exceeded in order for a single measurement to be considered real, as described by Weir [9]. The MD is calculated using the standard error of measurement (SEM), which is considered an absolute index of the reliability of a given test/measurement, not relative to the characteristics of the sample or population from which values were obtained. Unlike other reliability measures, such as the CV, the SEM and thus the MD, are not affected by between-subject variability [9]. If a subject’s measured values exceeded the MD, the change was considered a *true* change. Clinical significance at the individual level was reached when a score that exceeded the MD crossed the upper or lower limits of the accepted physiological range for each variable. For creatinine, this occurred in three subjects, one from G1 and two from G2, wherein values decreased pre to post, bringing them within the clinical reference range. For total cholesterol, changes observed in two subjects from G1 and one from G2 exceeded the MD. Specifically, the two subjects from G1 increased over time, moving from within range to out of range, while the subject from G2 decreased pre to post, entering the accepted reference range. Also worth noting is the fact that three individuals from the CRL group experienced changes in total cholesterol values that both exceeded the MD and moved in or out of range. In this case, one subject increased over time to leave the accepted range, one started outside of the range and remained out of range, and one decreased pre to post, entering back into range. All subjects remained within 3 standard deviations of the mean and exceeded the MD. Collectively, individual analysis supports the present hypothesis and also supports the notion of intra-subject diurnal variability. Furthermore, absolute monocytes and total cholesterol were distributed differently pre to post, increasing the probability for a type 1 statistical error [10].

These findings generally agree with previous literature. Aside from the research pertaining to PWS effects on performance [11-16], only a limited number of studies have also examined the clinical safety of PWS. Kedia et al. [17] looked at the effects of a multi-ingredient PWS containing caffeine, betaine, and dendrobium extract on body composition, performance measures, and hematological markers of clinical safety in healthy, young men and women undergoing concurrent resistance training for six weeks. While the investigators did not see an improvement in objective assessments of exercise performance or body composition with supplementation, they found the PWS to be well tolerated with no significant changes in clinical laboratory safety markers at the end of six weeks.

Similarly, Shelmadine et al. [18] examined the effects of 28 days consuming a commercially available PWS, NO-Shotgun®, combined with heavy resistance exercise on body composition, muscle strength and mass, myofibrillar protein content, markers of satellite cell activation, and clinical safety markers in male subjects. They found no negative side effects or abnormal impact on clinical safety markers after 28 days of supplementation. In a follow up study of the same nature, this time with a post-workout supplement added (NO-Synthesize®), Spillane et al. [19] again found no detrimental effects on clinical safety markers following 28 days supplementation and resistance training with NO-Shotgun®.

Farney et al. [20] investigated hemodynamic and hematological effects of two supplements containing caffeine and 1,3- dimethylamylamine (a constituent of geranium) after 14 days of supplementation in men and women, and found only a significant change in blood glucose for one of the supplements (Jack3d™) over this time period. A follow up to this study conducted by Whitehead et al. [21] supplemented with the same product containing caffeine and 1,3- dimethylamylamine (Jack3d™) over a 10-week period in healthy males and also found it did not negatively impact hematological markers of health when consumed daily.

Kendall et al. [22] investigated the safety and efficacy of a PWS containing caffeine, creatine, beta-alanine, amino acids and B-vitamins in recreationally trained, college-age men over an identical period of 28 days. In that study, no adverse effects were observed for renal or hepatic clinical blood markers or resting vital signs. Researchers concluded that PWS with similar ingredients in similar doses should be safe for ingestion periods up to 28 days in healthy males. More recently, Joy et al. [23] found that supplementation with a PWS containing caffeine, nitrate, and amino acids in healthy, recreationally active men and women was apparently safe when taken within recommended dosage guidelines for 28 days.

To our knowledge, this is the first study assessing the clinical safety of a PWS in an all-female population. Female-specific recommendations for sports nutrition and supplementation is an area that warrants more attention. A review article by Volek, Forsythe, and Kraemer [24], for instance, identifies the subtle, yet important differences in exercise metabolism between male and female athletes. The authors suggest that nutritional strategies, including nutrient timing and supplement use, should be tailored to meet the sex-specific needs of female athletes. In another review article addressing gender differences in sports nutrition, Tarnopolsky [25] similarly concluded that future studies in nutrition and metabolism should examine and consider sex differences in response to supplementation and exercise. It therefore seems prudent for future research to continue to address sports nutrition supplementation in females to evaluate both safety and efficacy in this population as compared to males.

### Limitations

The present study included a short duration supplementation period and small sample size. Future studies should examine the effects of supplementation for longer than 28 days among more subjects, especially given the fact that statistically significant interactions did take place over time in the present study. Again, while none of the significant variables left the accepted physiological range, the possibility that these could be the beginnings of adverse trends cannot be ruled out. This leaves long-term safety of PWS supplementation, at least greater than 28 days, still open to question.

## Conclusion

This study supports the hypothesis that a PWS containing caffeine, beta-alanine, and nitrate will not cause abnormal changes in hematological or clinical chemistry/metabolic markers, or resting vital signs among recreationally active females. Although there were statistically significant (p < 0.05) group by time interactions for absolute monocytes, MCH, creatinine, eGFR, and total cholesterol, all of the results remained well within accepted physiological ranges and were not clinically significant. In sum, it appears as though daily supplementation with up to 2 servings of the PWS used in this investigation, over a period of 28 days, had no adverse impact on markers of clinical safety among active adult females.

## Additional file

Additional file 1:
**Data collected pre and post supplementation.**

## Abbreviations

PWS: Pre-workout supplement(s)
BA: Beta-alanine
WBC: White blood cell
RBC: Red blood cell
MCV: Mean corpuscular volume
MCH: Mean corpuscular hemoglobin
MCHC: Mean corpuscular hemoglobin concentration
RDW: Red blood cell distribution width
BUN: Blood urea nitrogen
eGFR: Estimated glomerular filtration rate
AST: Aspartate aminotransferase
ALT: Alanine aminotransferase
CV: Coefficient of variation
MD: Minimum difference
SEM: Standard error of measurement

## References

[1] Kerksick C, Harvey T, Stout J, Campbell B, Wilborn C, Kreider R (2008). International society of sports nutrition position stand: nutrient timing. J Int Soc Sports Nutr.

[2] Eudy AE, Gordon LL, Hockaday BC, Lee DA, Lee V, Luu D (2013). Efficacy and safety of ingredients found in preworkout supplements. Am J Health Syst Pharm.

[3] Astorino TA, Roberson DW (2010). Efficacy of acute caffeine ingestion for short-term high-intensity exercise performance: a systematic review. J Strength Cond Res.

[4] Goldstein ER, Ziegenfuss T, Kalman D, Kreider R, Campbell B, Wilborn C (2010). International society of sports nutrition position stand: caffeine and performance. J Int Soc Sports Nutr.

[5] Graham TE (2001). Caffeine and exercise: metabolism, endurance and performance. Sports Med.

[6] Harris RC, Wise JA, Price KA, Kim HJ, Kim CK, Sale C (2012). Determinants of muscle carnosine content. Amino Acids.

[7] Derave W, Everaert I, Beeckman S, Baguet A (2010). Muscle carnosine metabolism and beta-alanine supplementation in relation to exercise and training. Sports Med.

[8] Quesnele JJ, Laframboise MA, Wong JJ, Kim P, Wells GD (2014). The effects of beta-alanine supplementation on performance: a systematic review of the literature. Int J Sport Nutr Exerc Metab.

[9] Weir JP (2005). Quantifying test-retest reliability using the intraclass correlation coefficient and the SEM. J Strength Cond Res.

[10] Delaney HD, Vargha A. The effect of nonnormality on student's two-sample *t* test. In: The education resources information center. U.S. Department of Education. 2000. http://eric.ed.gov/?q=ED443850&id=ED443850. Accessed 20 November 2014.

[11] Fukuda DH, Smith AE, Kendall KL, Stout JR (2010). The possible combinatory effects of acute consumption of caffeine, creatine, and amino acids on the improvement of anaerobic running performance in humans. Nutr Res.

[12] Hoffman JR, Kang J, Ratamess NA, Hoffman MW, Tranchina CP, Faigenbaum AD (2009). Examination of a pre-exercise, high energy supplement on exercise performance. J Int Soc Sports Nutr.

[13] Lowery RP, Joy JM, Dudeck JE, Oliveira de Souza E, McCleary SA, Wells S (2013). Effects of 8 weeks of Xpand(R) 2X pre workout supplementation on skeletal muscle hypertrophy, lean body mass, and strength in resistance trained males. J Int Soc Sports Nutr.

[14] Outlaw JJ, Wilborn CD, Smith-Ryan AE, Hayward SE, Urbina SL, Taylor LW (2014). Acute effects of a commercially-available pre-workout supplement on markers of training: a double-blind study. J Int Soc Sports Nutr.

[15] Smith AE, Fukuda DH, Kendall KL, Stout JR (2010). The effects of a pre-workout supplement containing caffeine, creatine, and amino acids during three weeks of high-intensity exercise on aerobic and anaerobic performance. J Int Soc Sports Nutr.

[16] Spradley BD, Crowley KR, Tai CY, Kendall KL, Fukuda DH, Esposito EN (2012). Ingesting a pre-workout supplement containing caffeine, B-vitamins, amino acids, creatine, and beta-alanine before exercise delays fatigue while improving reaction time and muscular endurance. Nutr Metab (Lond).

[17] Kedia AW, Hofheins JE, Habowski SM, Ferrando AA, Gothard MD, Lopez HL (2014). Effects of a pre-workout supplement on lean mass, muscular performance, subjective workout experience and biomarkers of safety. Int J Med Sci.

[18] Shelmadine B, Cooke M, Buford T, Hudson G, Redd L, Leutholtz B (2009). Effects of 28 days of resistance exercise and consuming a commercially available pre-workout supplement, NO-Shotgun(R), on body composition, muscle strength and mass, markers of satellite cell activation, and clinical safety markers in males. J Int Soc Sports Nutr.

[19] Spillane M, Schwarz N, Leddy S, Correa T, Minter M, Longoria V (2011). Effects of 28 days of resistance exercise while consuming commercially available pre- and post-workout supplements, NO-Shotgun(R) and NO-Synthesize(R) on body composition, muscle strength and mass, markers of protein synthesis, and clinical safety markers in males. Nutr Metab (Lond).

[20] Farney TM, McCarthy CG, Canale RE, Allman RJ, Bloomer RJ (2012). Hemodynamic and hematologic profile of healthy adults ingesting dietary supplements containing 1,3-dimethylamylamine and caffeine. Nutr Metab Insights.

[21] Whitehead PN, Schilling BK, Farney TM, Bloomer RJ (2012). Impact of a dietary supplement containing 1,3-dimethylamylamine on blood pressure and bloodborne markers of health: a 10-week intervention study. Nutr Metab Insights.

[22] Kendall KL, Moon JR, Fairman CM, Spradley BD, Tai C-Y, Falcone PH (2014). Ingesting a preworkout supplement containing caffeine, creatine, beta-alanine, amino acids, and B vitamins for 28 days is both safe and efficacious in recreationally active men. Nutr Res.

[23] Joy JM, Mosman MM, Falcone PH, Tai C-Y, Carson LR, Kimber D (2014). Safety of 28 days consumption of a pre-workout supplement. J Int Soc Sports Nutr.

[24] Volek JS, Forsythe CE, Kraemer WJ (2006). Nutritional aspects of women strength athletes. Br J Sports Med.

[25] Tarnopolsky MA (2000). Gender differences in metabolism; nutrition and supplements. J Sci Med Sport.

